# 2-Amino­terephthalic acid *N*,*N*-dimethyl­formamide disolvate

**DOI:** 10.1107/S1600536812031431

**Published:** 2012-07-21

**Authors:** Stefan Loos, Wilhelm Seichter, Edwin Weber, Florian Mertens

**Affiliations:** a Institut für Physikalische Chemie, Leipziger Strasse 29, 09596 Freiberg, Germany; b Institut für Organische Chemie, Leipziger Strasse 29, 09596 Freiberg, Germany

## Abstract

The asymmetric unit of the title structure, C_8_H_7_NO_4_·2C_3_H_7_NO, contains one 2-amino­terephthalic acid and two *N*,*N*-dimethyl­formamide mol­ecules. Strong O—H⋯O hydrogen bonds between the acidic carb­oxy H atoms of 2-am­ino­terephthalic acid and the O atoms of both solvent mol­ecules form linear 1:2 complex units. One H atom of the amine group is involved in intra­molecular N—H⋯O hydrogen bonding, whereas the second one takes part in an inter­molecular N—H⋯O connection. Furthermore, the crystal is stabilized by weak C—H⋯O hydrogen bonds.

## Related literature

For the structure of 2-amino­terephthalic acid dimethyl ester, see: Brüning *et al.* (2009 ▶). For the use of this carb­oxy­lic acid in the synthesis of porous structures, see: Bauer *et al.* (2008 ▶). For a co-crystal of 2-amino­terephthalic acid, see: Xiao *et al.* (2011 ▶).
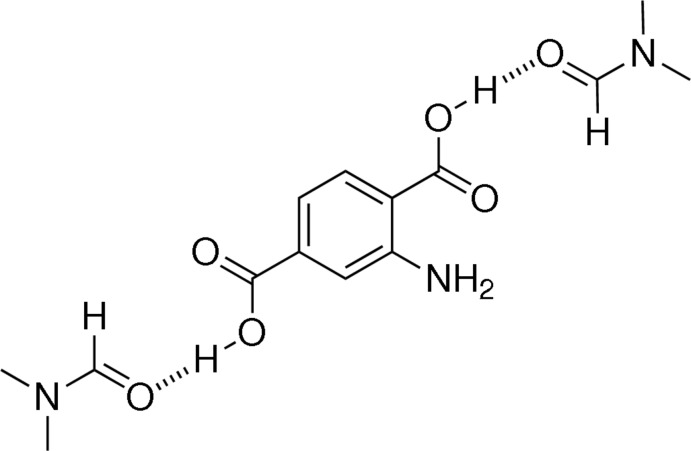


## Experimental

### 

#### Crystal data

C_8_H_7_NO_4_·2C_3_H_7_NO
*M* *_r_* = 327.34
Monoclinic, 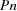

*a* = 7.8393 (2) Å
*b* = 9.7462 (2) Å
*c* = 10.9147 (2) Å
β = 103.251 (1)°
*V* = 811.72 (3) Å^3^
*Z* = 2
Mo *K*α radiation
μ = 0.11 mm^−1^
*T* = 153 K
0.58 × 0.51 × 0.37 mm

#### Data collection

Bruker APEXII CCD area-detector diffractometer
Absorption correction: multi-scan (*SADABS*; Bruker, 2007 ▶) *T*_min_ = 0.941, *T*_max_ = 0.962
19574 measured reflections
2260 independent reflections
2157 reflections with *I* > 2σ(*I*)
*R*_int_ = 0.020

#### Refinement

*R*[*F* ^2^ > 2σ(*F* ^2^)] = 0.031
*wR*(*F* ^2^) = 0.092
*S* = 1.05
2260 reflections
223 parameters
4 restraints
H atoms treated by a mixture of independent and constrained refinement
Δρ_max_ = 0.21 e Å^−3^
Δρ_min_ = −0.17 e Å^−3^

### 

Data collection: *APEX2* (Bruker, 2007 ▶); cell refinement: *SAINT-NT* (Bruker, 2007 ▶); data reduction: *SAINT-NT*; program(s) used to solve structure: *SHELXS97* (Sheldrick, 2008 ▶); program(s) used to refine structure: *SHELXL97* (Sheldrick, 2008 ▶); molecular graphics: *ORTEP-3* (Farrugia, 1997 ▶); software used to prepare material for publication: *SHELXTL* (Sheldrick, 2008 ▶).

## Supplementary Material

Crystal structure: contains datablock(s) I, global. DOI: 10.1107/S1600536812031431/fy2048sup1.cif

Structure factors: contains datablock(s) I. DOI: 10.1107/S1600536812031431/fy2048Isup2.hkl

Supplementary material file. DOI: 10.1107/S1600536812031431/fy2048Isup3.cdx

Supplementary material file. DOI: 10.1107/S1600536812031431/fy2048Isup4.cml

Additional supplementary materials:  crystallographic information; 3D view; checkCIF report

## Figures and Tables

**Table 1:** Hydrogen-bond geometry (Å, °)

*D*—H⋯*A*	*D*—H	H⋯*A*	*D*⋯*A*	*D*—H⋯*A*
N1—H1*A*⋯O1	0.81 (3)	2.03 (3)	2.685 (2)	138 (3)
N1—H1*B*⋯O4^i^	0.84 (2)	2.14 (3)	2.960 (2)	169 (3)
O3—H3⋯O1*A* ^ii^	0.84	1.72	2.5549 (19)	173
O2—H2⋯O1*B*	0.84	1.74	2.577 (2)	174
C1*A*—H1*AA*⋯O4^iii^	0.95	2.50	3.219 (2)	133
C1*B*—H1*BA*⋯O1	0.95	2.42	3.155 (2)	134
C3—H3*A*⋯O4^i^	0.95	2.57	3.344 (2)	139

Symmetry codes: (i) 
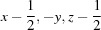
; (ii) 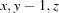; (iii) 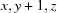.

**Fig. 1.**
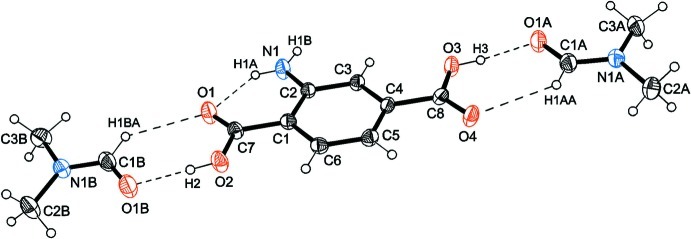
ORTEP-plot of the title compound. Ellipsoids are shown at the 50% probability level.

**Fig. 2.**
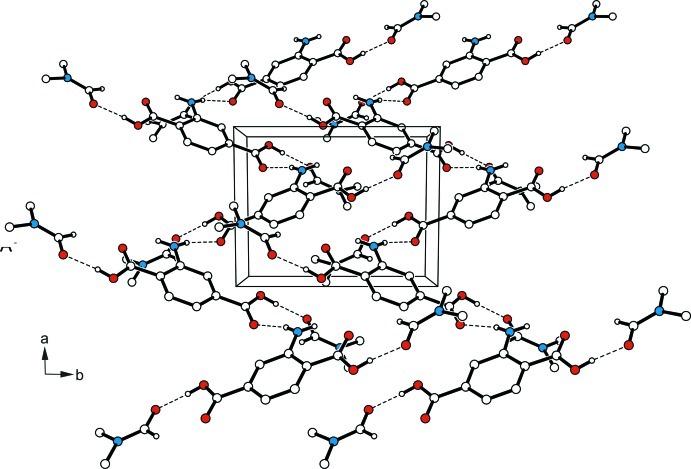
Packing diagram of the title compound. Intermoelcular hydrogen bonds are shown as dashed lines. Atom colours: red: oxygen, white (small): hydrogen, blue: nitrogen, white (large): carbon.

## supplementary crystallographic information

### Comment

In recent years, carboxylic acids became of great interest concerning the synthesis of porous metal oragnic framework compounds, often using *N*,*N*-dimethylformamide as an apropriate solvent. The knowledge of the behavior of the organic linker molecule in the solvent can provide important information about the acidic character and hence the synthesis strategy. Analysing the interaction of the linker molecule with the solvent in the crystal, we can get insight in the structure of the carboxylic acid in solution (DMF). The crystal structure composed of 2-aminoterephthalic acid and *N*,*N*-dimethylformamide has monoclinic symmetry (space group: *Pn*). It is characterised by strong hydrogen bonding between the acidic carboxy H-atoms of 2-aminoterephthalic acid and the O-atoms of the solvent molecules [O2—H2···O1B 1.74 Å, 174 °, O3—H3···O1A 1.742 Å, 173 °]. One hydrogen of the amino group is involved in intramolecular hydrogen bonding [N1—H1A···O1 2.02 (2) Å, 138 (3) °] whereas the second one takes part in intermolecular connection [N1—H2···H1B···O4 2.14 (3) Å, 168 (3) °]. Furthermore, the crystal is stabilized by weak hydrogen bonds of the C—H···O type [*d*(H···O) = 2.42–2.57 Å]. Due to the given mode of non-covalent intermolecular bonding the crystal structure is composed of linear 1:2 complex units which are associated among one another by N—H···O interactions.

### Experimental

2-Aminoterephthalic acid (>99%) was purchased from Sigma Aldrich and used without further purification. Crystals of 2-aminoterephthalic acid in *N*,*N*-dimethylformamide were obtained by the partial dissolution of 300 mg (1.65 mmol) 2-aminoterephthalic acid in 50 ml acetone and subsequent addition of the appropriate amount of *N*,*N*-dimethylfromamide (10 ml, 130 mmol) to completely dissolve the carboxylic acid. Afterwards, acetone was slowly evaporated from the solution. Colourless crystals of 2-aminoterephthalic acid * 2 *N*,*N*-dimethylformamide crystallised from the solution after removal of acetone.

### Refinement

The positions of the amino hydrogens H1A and H1B could be obtained from the difference electron-density map. Lengths of the N-H bonds were restrained to a target value of 0.84 (1) Å. The carbon- and oxygen-bound H atoms were placed in calculated positions and were treated as riding on the parent C and O atoms with O-H = 0.84, C-H(aryl)/C-H(formyl) = 0.95 and C-H(methyl) = 0.98 Å; U_iso_(H) = k U_eq_(C,O), where k = 1.5 for methyl and hydroxyl and k = 1.2 for all other H-atoms.

The large s.u. on the Flack parameter suggested averaging of Friedel pairs. To average Friedel opposites, we used the MERG 4 command in SHELXL97 in the final refinement step.

### Crystal data

**Table:**

C_8_H_7_NO_4_·2C_3_H_7_NO	*F*(000) = 348
*M**_r_* = 327.34	*D*_x_ = 1.339 Mg m^−^^3^
Monoclinic, *P**n*	Mo *K*α radiation, λ = 0.71073 Å
Hall symbol: P -2yac	Cell parameters from 9996 reflections
*a* = 7.8393 (2) Å	θ = 2.8–33.3°
*b* = 9.7462 (2) Å	µ = 0.11 mm^−^^1^
*c* = 10.9147 (2) Å	*T* = 153 K
β = 103.251 (1)°	Irregular, colourless
*V* = 811.72 (3) Å^3^	0.58 × 0.51 × 0.37 mm
*Z* = 2	

### Data collection

**Table:**

Bruker APEXII CCD area-detector diffractometer	2260 independent reflections
Radiation source: fine-focus sealed tube	2157 reflections with *I* > 2σ(*I*)
Graphite monochromator	*R*_int_ = 0.020
φ and ω scans	θ_max_ = 29.5°, θ_min_ = 2.1°
Absorption correction: multi-scan (*SADABS*; Bruker, 2007)	*h* = −10→10
*T*_min_ = 0.941, *T*_max_ = 0.962	*k* = −13→13
19574 measured reflections	*l* = −15→15

### Refinement

**Table:**

Refinement on *F*^2^	Primary atom site location: structure-invariant direct methods
Least-squares matrix: full	Secondary atom site location: difference Fourier map
*R*[*F*^2^ > 2σ(*F*^2^)] = 0.031	Hydrogen site location: inferred from neighbouring sites
*wR*(*F*^2^) = 0.092	H atoms treated by a mixture of independent and constrained refinement
*S* = 1.05	*w* = 1/[σ^2^(*F*_o_^2^) + (0.0565*P*)^2^ + 0.133*P*] where *P* = (*F*_o_^2^ + 2*F*_c_^2^)/3
2260 reflections	(Δ/σ)_max_ < 0.001
223 parameters	Δρ_max_ = 0.21 e Å^−^^3^
4 restraints	Δρ_min_ = −0.17 e Å^−^^3^

### Special details

**Table:**

Geometry. All e.s.d.'s (except the e.s.d. in the dihedral angle between two l.s. planes) are estimated using the full covariance matrix. The cell e.s.d.'s are taken into account individually in the estimation of e.s.d.'s in distances, angles and torsion angles; correlations between e.s.d.'s in cell parameters are only used when they are defined by crystal symmetry. An approximate (isotropic) treatment of cell e.s.d.'s is used for estimating e.s.d.'s involving l.s. planes.
Refinement. Refinement of *F*^2^ against ALL reflections. The weighted *R*-factor *wR* and goodness of fit *S* are based on *F*^2^, conventional *R*-factors *R* are based on *F*, with *F* set to zero for negative *F*^2^. The threshold expression of *F*^2^ > σ(*F*^2^) is used only for calculating *R*-factors(gt) *etc*. and is not relevant to the choice of reflections for refinement. *R*-factors based on *F*^2^ are statistically about twice as large as those based on *F*, and *R*- factors based on ALL data will be even larger.

### Fractional atomic coordinates and isotropic or equivalent isotropic displacement parameters (Å^2^)

**Table:**

	*x*	*y*	*z*	*U*_iso_*/*U*_eq_	
O1	0.25655 (18)	0.55493 (13)	0.50464 (13)	0.0305 (3)	
O2	0.43465 (18)	0.57438 (14)	0.69541 (12)	0.0288 (3)	
H2	0.3800	0.6484	0.6961	0.043*	
O3	0.55866 (19)	−0.12115 (13)	0.49500 (12)	0.0293 (3)	
H3	0.6119	−0.1963	0.4984	0.044*	
O4	0.72462 (18)	−0.09748 (14)	0.69012 (12)	0.0289 (3)	
N1	0.2490 (2)	0.31517 (16)	0.38262 (14)	0.0282 (3)	
H1A	0.214 (3)	0.3941 (14)	0.385 (3)	0.035 (6)*	
H1B	0.228 (4)	0.259 (3)	0.323 (2)	0.057 (9)*	
C1	0.4280 (2)	0.36183 (18)	0.59253 (14)	0.0197 (3)	
C2	0.3678 (2)	0.27585 (15)	0.48708 (14)	0.0188 (3)	
C3	0.4335 (2)	0.14046 (16)	0.49115 (14)	0.0197 (3)	
H3A	0.3961	0.0818	0.4206	0.024*	
C4	0.5515 (2)	0.09231 (17)	0.59659 (14)	0.0196 (3)	
C5	0.6101 (2)	0.17684 (18)	0.70191 (15)	0.0227 (3)	
H5	0.6911	0.1433	0.7742	0.027*	
C6	0.5478 (2)	0.30979 (17)	0.69856 (15)	0.0224 (3)	
H6	0.5868	0.3675	0.7696	0.027*	
C7	0.3648 (2)	0.50511 (16)	0.59147 (16)	0.0215 (3)	
C8	0.6191 (2)	−0.05168 (17)	0.59927 (15)	0.0217 (3)	
O1A	0.7117 (2)	0.64886 (15)	0.48597 (13)	0.0359 (3)	
N1A	0.8832 (2)	0.48490 (17)	0.59960 (15)	0.0275 (3)	
C1A	0.8124 (3)	0.60873 (19)	0.58446 (17)	0.0288 (4)	
H1AA	0.8403	0.6708	0.6532	0.035*	
C2A	0.9926 (3)	0.4434 (2)	0.7207 (2)	0.0346 (4)	
H2A1	0.9982	0.5183	0.7815	0.052*	
H2A2	1.1110	0.4224	0.7109	0.052*	
H2A3	0.9421	0.3618	0.7512	0.052*	
C3A	0.8513 (3)	0.3853 (2)	0.49843 (19)	0.0342 (4)	
H3A1	0.7960	0.4309	0.4192	0.051*	
H3A2	0.7736	0.3132	0.5166	0.051*	
H3A3	0.9628	0.3446	0.4910	0.051*	
O1B	0.2883 (2)	0.80983 (15)	0.70391 (14)	0.0362 (3)	
N1B	0.10043 (19)	0.96613 (16)	0.59172 (15)	0.0258 (3)	
C1B	0.1865 (2)	0.84831 (19)	0.60504 (18)	0.0284 (4)	
H1BA	0.1694	0.7890	0.5342	0.034*	
C2B	0.1191 (3)	1.0619 (2)	0.6961 (2)	0.0347 (4)	
H2B1	0.1608	1.0127	0.7756	0.052*	
H2B2	0.0053	1.1041	0.6951	0.052*	
H2B3	0.2037	1.1334	0.6878	0.052*	
C3B	−0.0088 (3)	1.0077 (2)	0.47142 (19)	0.0334 (4)	
H3B1	0.0445	1.0868	0.4392	0.050*	
H3B2	−0.1254	1.0328	0.4823	0.050*	
H3B3	−0.0190	0.9315	0.4115	0.050*	

### Atomic displacement parameters (Å^2^)

**Table:**

	*U*^11^	*U*^22^	*U*^33^	*U*^12^	*U*^13^	*U*^23^
O1	0.0356 (7)	0.0217 (6)	0.0287 (7)	0.0068 (5)	−0.0045 (5)	−0.0052 (5)
O2	0.0347 (7)	0.0224 (6)	0.0248 (6)	0.0032 (5)	−0.0028 (5)	−0.0082 (5)
O3	0.0399 (7)	0.0205 (6)	0.0232 (6)	0.0087 (5)	−0.0018 (5)	−0.0003 (5)
O4	0.0360 (7)	0.0214 (6)	0.0246 (6)	0.0041 (5)	−0.0028 (5)	0.0039 (5)
N1	0.0353 (8)	0.0219 (6)	0.0209 (6)	0.0074 (6)	−0.0069 (5)	−0.0031 (5)
C1	0.0210 (7)	0.0202 (8)	0.0167 (6)	−0.0003 (5)	0.0018 (5)	−0.0022 (5)
C2	0.0206 (7)	0.0179 (7)	0.0167 (6)	0.0009 (5)	0.0019 (5)	−0.0005 (5)
C3	0.0224 (7)	0.0186 (7)	0.0161 (6)	0.0004 (5)	0.0002 (5)	−0.0003 (5)
C4	0.0229 (7)	0.0167 (7)	0.0185 (7)	0.0016 (5)	0.0032 (6)	0.0020 (5)
C5	0.0247 (7)	0.0239 (8)	0.0171 (6)	0.0015 (6)	−0.0002 (6)	0.0011 (6)
C6	0.0246 (7)	0.0236 (7)	0.0177 (7)	−0.0001 (6)	0.0023 (6)	−0.0029 (6)
C7	0.0237 (7)	0.0181 (7)	0.0222 (7)	−0.0007 (6)	0.0041 (6)	−0.0045 (6)
C8	0.0244 (8)	0.0207 (8)	0.0191 (7)	−0.0002 (6)	0.0031 (6)	0.0033 (6)
O1A	0.0498 (9)	0.0278 (7)	0.0260 (7)	0.0144 (6)	−0.0001 (6)	−0.0016 (5)
N1A	0.0289 (8)	0.0272 (8)	0.0258 (7)	0.0055 (6)	0.0050 (6)	0.0009 (6)
C1A	0.0348 (9)	0.0243 (8)	0.0262 (8)	0.0052 (7)	0.0046 (7)	−0.0012 (6)
C2A	0.0313 (9)	0.0363 (10)	0.0340 (10)	0.0089 (8)	0.0030 (8)	0.0063 (8)
C3A	0.0427 (11)	0.0266 (9)	0.0341 (10)	0.0100 (8)	0.0104 (8)	−0.0024 (7)
O1B	0.0456 (8)	0.0271 (7)	0.0320 (7)	0.0090 (6)	0.0007 (6)	−0.0055 (5)
N1B	0.0258 (7)	0.0237 (7)	0.0268 (7)	0.0012 (6)	0.0036 (6)	−0.0039 (6)
C1B	0.0334 (9)	0.0226 (8)	0.0288 (9)	−0.0008 (7)	0.0065 (7)	−0.0060 (6)
C2B	0.0369 (10)	0.0308 (9)	0.0333 (9)	0.0062 (7)	0.0015 (8)	−0.0121 (8)
C3B	0.0313 (9)	0.0395 (11)	0.0276 (9)	0.0061 (8)	0.0031 (7)	−0.0006 (8)

### Geometric parameters (Å, º)

**Table:**

O1—C7	1.219 (2)	N1A—C1A	1.323 (2)
O2—C7	1.326 (2)	N1A—C3A	1.448 (3)
O2—H2	0.8400	N1A—C2A	1.458 (2)
O3—C8	1.316 (2)	C1A—H1AA	0.9500
O3—H3	0.8400	C2A—H2A1	0.9800
O4—C8	1.221 (2)	C2A—H2A2	0.9800
N1—C2	1.352 (2)	C2A—H2A3	0.9800
N1—H1A	0.820 (10)	C3A—H3A1	0.9800
N1—H1B	0.837 (10)	C3A—H3A2	0.9800
C1—C6	1.407 (2)	C3A—H3A3	0.9800
C1—C2	1.414 (2)	O1B—C1B	1.244 (2)
C1—C7	1.481 (2)	N1B—C1B	1.323 (2)
C2—C3	1.413 (2)	N1B—C3B	1.451 (3)
C3—C4	1.383 (2)	N1B—C2B	1.454 (2)
C3—H3A	0.9500	C1B—H1BA	0.9500
C4—C5	1.403 (2)	C2B—H2B1	0.9800
C4—C8	1.498 (2)	C2B—H2B2	0.9800
C5—C6	1.382 (2)	C2B—H2B3	0.9800
C5—H5	0.9500	C3B—H3B1	0.9800
C6—H6	0.9500	C3B—H3B2	0.9800
O1A—C1A	1.242 (2)	C3B—H3B3	0.9800
			
C7—O2—H2	109.5	O1A—C1A—H1AA	117.9
C8—O3—H3	109.5	N1A—C1A—H1AA	117.9
C2—N1—H1A	114 (2)	N1A—C2A—H2A1	109.5
C2—N1—H1B	116 (2)	N1A—C2A—H2A2	109.5
H1A—N1—H1B	130 (3)	H2A1—C2A—H2A2	109.5
C6—C1—C2	119.41 (14)	N1A—C2A—H2A3	109.5
C6—C1—C7	120.36 (14)	H2A1—C2A—H2A3	109.5
C2—C1—C7	120.23 (14)	H2A2—C2A—H2A3	109.5
N1—C2—C3	117.82 (14)	N1A—C3A—H3A1	109.5
N1—C2—C1	123.73 (14)	N1A—C3A—H3A2	109.5
C3—C2—C1	118.44 (14)	H3A1—C3A—H3A2	109.5
C4—C3—C2	120.82 (14)	N1A—C3A—H3A3	109.5
C4—C3—H3A	119.6	H3A1—C3A—H3A3	109.5
C2—C3—H3A	119.6	H3A2—C3A—H3A3	109.5
C3—C4—C5	120.90 (15)	C1B—N1B—C3B	121.47 (16)
C3—C4—C8	120.03 (14)	C1B—N1B—C2B	120.93 (16)
C5—C4—C8	119.07 (14)	C3B—N1B—C2B	117.53 (15)
C6—C5—C4	118.77 (14)	O1B—C1B—N1B	124.52 (17)
C6—C5—H5	120.6	O1B—C1B—H1BA	117.7
C4—C5—H5	120.6	N1B—C1B—H1BA	117.7
C5—C6—C1	121.65 (15)	N1B—C2B—H2B1	109.5
C5—C6—H6	119.2	N1B—C2B—H2B2	109.5
C1—C6—H6	119.2	H2B1—C2B—H2B2	109.5
O1—C7—O2	122.68 (15)	N1B—C2B—H2B3	109.5
O1—C7—C1	123.64 (14)	H2B1—C2B—H2B3	109.5
O2—C7—C1	113.67 (15)	H2B2—C2B—H2B3	109.5
O4—C8—O3	123.85 (16)	N1B—C3B—H3B1	109.5
O4—C8—C4	121.95 (15)	N1B—C3B—H3B2	109.5
O3—C8—C4	114.17 (14)	H3B1—C3B—H3B2	109.5
C1A—N1A—C3A	121.45 (17)	N1B—C3B—H3B3	109.5
C1A—N1A—C2A	120.58 (16)	H3B1—C3B—H3B3	109.5
C3A—N1A—C2A	117.94 (16)	H3B2—C3B—H3B3	109.5
O1A—C1A—N1A	124.11 (17)		
			
C6—C1—C2—N1	177.95 (16)	C6—C1—C7—O1	−177.30 (17)
C7—C1—C2—N1	−1.7 (2)	C2—C1—C7—O1	2.4 (3)
C6—C1—C2—C3	−1.3 (2)	C6—C1—C7—O2	1.5 (2)
C7—C1—C2—C3	179.05 (15)	C2—C1—C7—O2	−178.78 (15)
N1—C2—C3—C4	−178.14 (15)	C3—C4—C8—O4	179.29 (16)
C1—C2—C3—C4	1.1 (2)	C5—C4—C8—O4	−0.5 (2)
C2—C3—C4—C5	−0.5 (2)	C3—C4—C8—O3	1.0 (2)
C2—C3—C4—C8	179.80 (15)	C5—C4—C8—O3	−178.70 (15)
C3—C4—C5—C6	−0.1 (2)	C3A—N1A—C1A—O1A	1.7 (3)
C8—C4—C5—C6	179.68 (16)	C2A—N1A—C1A—O1A	−176.6 (2)
C4—C5—C6—C1	−0.1 (3)	C3B—N1B—C1B—O1B	176.51 (18)
C2—C1—C6—C5	0.8 (2)	C2B—N1B—C1B—O1B	−0.4 (3)
C7—C1—C6—C5	−179.54 (16)		

### Hydrogen-bond geometry (Å, º)

**Table:**

*D*—H···*A*	*D*—H	H···*A*	*D*···*A*	*D*—H···*A*
N1—H1*A*···O1	0.81 (3)	2.03 (3)	2.685 (2)	138 (3)
N1—H1*B*···O4^i^	0.84 (2)	2.14 (3)	2.960 (2)	169 (3)
O3—H3···O1*A*^ii^	0.84	1.72	2.5549 (19)	173
O2—H2···O1*B*	0.84	1.74	2.577 (2)	174
C1*A*—H1*AA*···O4^iii^	0.95	2.50	3.219 (2)	133
C1*B*—H1*BA*···O1	0.95	2.42	3.155 (2)	134
C3—H3*A*···O4^i^	0.95	2.57	3.344 (2)	139

Symmetry codes: (i) *x*−1/2, −*y*, *z*−1/2; (ii) *x*, *y*−1, *z*; (iii) *x*, *y*+1, *z*.
